# The 1β-Hydroxy-Deoxycholic Acid to Deoxycholic Acid Urinary Metabolic Ratio: Toward a Phenotyping of CYP3A Using an Endogenous Marker?

**DOI:** 10.3390/jpm11020150

**Published:** 2021-02-20

**Authors:** Gaëlle Magliocco, Jules Desmeules, Marija Bosilkovska, Aurélien Thomas, Youssef Daali

**Affiliations:** 1 Division of Clinical Pharmacology and Toxicology, Geneva University Hospitals, 1205 Geneva, Switzerland; gaelle.magliocco@unige.ch (G.M.); jules.desmeules@hcuge.ch (J.D.); marija.bosilkovska@hcuge.ch (M.B.); 2 Institute of Pharmaceutical Sciences of Western Switzerland, University of Geneva, 1206 Geneva, Switzerland; 3 Swiss Center for Applied Human Toxicology, 1205 Geneva, Switzerland; aurelien.thomas@chuv.ch; 4 Faculty of Medicine, University of Geneva, 1206 Geneva, Switzerland; 5 Forensic Toxicology and Chemistry Unit, CURML, 1000 Lausanne University Hospital, Geneva University Hospitals, Lausanne, 1205 Geneva, Switzerland; 6 Faculty Unit of Toxicology, CURML, Faculty of Biology and Medicine, University of Lausanne, 1000 Lausanne, Switzerland

**Keywords:** CYP450, phenotyping, CYP3A, bile acid, biomarker

## Abstract

In this study, we assessed the potential use of the 1β-hydroxy-deoxycholic acid (1β-OH-DCA) to deoxycholic acid (DCA) urinary metabolic ratio (UMR) as a CYP3A metric in ten male healthy volunteers. Midazolam (MDZ) 1 mg was administered orally at three sessions: alone (control session), after pre-treatment with fluvoxamine 50 mg (12 h and 2 h prior to MDZ administration), and voriconazole 400 mg (2 h before MDZ administration) (inhibition session), and after a 7-day pre-treatment with the inducer rifampicin 600 mg (induction session). The 1β-OH-DCA/DCA UMR was measured at each session, and correlations with MDZ metrics were established. At baseline, the 1β-OH-DCA/DCA UMR correlated significantly with oral MDZ clearance (*r* = 0.652, *p* = 0.041) and C_max_ (*r* = −0.652, *p* = 0.041). In addition, the modulation of CYP3A was reflected in the 1β-OH-DCA/DCA UMR after the intake of rifampicin (induction ratio = 11.4, *p* < 0.01). During the inhibition session, a non-significant 22% decrease in 1β-OH-DCA/DCA was observed (*p* = 0.275). This result could be explained by the short duration of CYP3A inhibitors intake fixed in our clinical trial. Additional studies, particularly involving CYP3A inhibition for a longer period and larger sample sizes, are needed to confirm the 1β-OH-DCA/DCA metric as a suitable CYP3A biomarker.

## 1. Introduction

Macrolide antibiotics, calcium channel blockers, immunosuppressants, benzodiazepines, and many other compounds are metabolized by the cytochrome P450 3A (CYP3A) enzymes [1]. It is estimated that approximately 30% of all marketed drugs are eliminated through this subfamily [1]. The metabolism of CYP3A substrates may be largely affected by the important pharmacokinetic variability of these enzymes’ function, leading to serious therapeutic complications [2,3]. Drug–drug interactions are a major cause of variability in CYP3A activity [1]. CYP3A can be induced by ligands that bind to the nuclear receptors PXR and CAR, reducing the drug levels [4]. In contrast, a number of endogenous and exogenous compounds can also inhibit CYP3A activity, increasing the drug concentrations [5]. For example, the co-administration of CYP3A inhibitors, such as diltiazem, clarithromycin, or amiodarone, with a statin may cause rhabdomyolysis [6,7,8]. Genetic polymorphisms can also strongly influence CYP3A5 activity. In particular, CYP3A5*3, resulting in a non-functional protein, is the most frequent variant allele of CYP3A5 [3,9]. Conversely, few polymorphisms have been described for CYP3A4 [9].

To date, the standard approach for measuring CYP3A activity is phenotyping using a probe drug, taking into account the environmental component that explains most of the enzymatic variability [10].

One of the first tests developed to assess CYP3A activity was the erythromycin breath test. After the administration of [^14^C-N-methyl]-erythromycin, the amount of exhaled ^14^CO2 is measured, allowing prediction of CYP3A activity [11]. However, the presence of radiolabeled ^14^C makes its use in clinical practice more complex [12]. In addition, erythromycin lacks specificity, as it is also a P-glycoprotein substrate [11].

Currently, midazolam (MDZ) is considered as the gold standard for measuring CYP3A activity. The biotransformation of MDZ into 1′-hydroxy-midazolam (OH-MDZ) is mediated exclusively through CYP3A, and MDZ is not a substrate for the main drug transporters [13]. However, phenotyping using MDZ requires the administration of an exogenous substance, which is not always well received by patients and not completely risk-free [14].

CYP3A enzymes are known to metabolize endogenous sterol lipids, including cortisol, cortisone, and dehydroepiandrosterone [15]. The metabolism of cholesterol into 4β-hydroxy-cholesterol has also been described as an endogenous CYP3A probe, but the long half-life of this latter may, to some extent, affect its use for the detection of rapid changes in CYP3A activity [16]. More recently, five acyl-carnitines derivatives have been identified as CYP3A biomarkers using untargeted metabolomics [17]. Several multivariate equations using one or more of these markers described above have been developed to optimize the prediction of CYP3A activity through MDZ clearance (CL) [15]. At baseline, an equation using 6β-hydroxy-cortisol to cortisol and 6β-hydroxy-cortisone to cortisone urinary metabolic ratios as well as sex as covariates explains 26.1% of the variation in MDZ CL [18].

The detoxification of bile acids is primarily regulated by the CYP3A4 enzyme in humans. This enzyme protects against the cytotoxicity of bile acids exacerbated at high concentration levels [19,20]. In fact, rifampicin, a strong inducer of CYP3A, can be used to treat some cholestatic conditions via the stimulation of the hydroxylation of bile acids [20]. Several years ago, Bodin et al. [20] observed significant amounts of 1β-hydroxy-deoxycholic acid (1β-OH-DCA), a secondary bile acid, in the urine samples from five patients treated with carbamazepine compared to ten healthy volunteers. Then, it was demonstrated by Hayes et al. [21] that deoxycholic acid (DCA) is specifically metabolized into 1β-OH-DCA by CYP3A4 and CYP3A7 using recombinant human CYP450 enzymes. In contrast, CYP3A5 isoenzyme seems not or poorly involved in the 1β-hydroxylation of DCA (around 10–50 times lower) [21,22,23]. DCA and 1β-OH-DCA can be conjugated to glycine, taurine, glucuronic acid, and sulfate in human hepatocytes. For both bile acids, the glycine conjugate appears to be the major metabolite, as reported by Hayes et al. [21]. In the study of Hayes et al. [21], they also measured the 1β-OH-DCA to DCA urinary metabolic ratio (UMR) in a sample from a carbamazepine-treated patient that was 7-fold higher than that of a control sample (pooled urine from six healthy controls). The authors concluded that further studies should be conducted to evaluate the promising ability of the 1β-OH-DCA/DCA UMR to phenotype the CYP3A4 enzyme.

Therefore, the objective of our work was to provide additional data concerning the couple 1β-OH-DCA/DCA in human urine. The study consisted of analyzing urine samples from ten healthy male volunteers receiving oral MDZ (as part of a phenotyping cocktail) alone, after inhibition, and after the induction of CYP3A, in order to measure the impact on the 1β-OH-DCA/DCA ratio and to verify the correlation with MDZ, which is the model substrate drug for CYP3A activity.

## 2. Materials and Methods

### 2.1. Study Design and Population

Data and samples were extracted from another study involving ten healthy male volunteers, with the primary objective of evaluating whether the capillary dried blood spot sampling method was appropriate for the simultaneous phenotyping of CYP450 and P-glycoprotein using a cocktail approach. More details about the study have been described elsewhere [24].

All the subjects were non-smokers, had normal liver function tests, and were not taking any drugs that affect CYP450 activity. In addition, they were not allowed to drink grapefruit juice for at least one week before and for the duration of the clinical trial.

**Figure 1:**
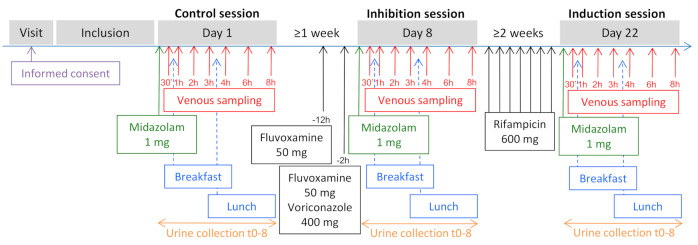
Study timeline. Midazolam 1 mg, as part of the Geneva cocktail, was orally administered on days 1, 8, and 22. Fluvoxamine 50 mg was administered 12 h and 2 h before the administration of midazolam (inhibition session). Voriconazole 400 mg was administered 2 h before the administration of midazolam (inhibition session). Rifampicin 600 mg was administered once daily for seven days until the evening before the administration of midazolam (induction session).

Figure 1 describes the study timeline for this sub-study.

Healthy volunteers were administered, as part of the Geneva cocktail, MDZ 1 mg for CYP3A phenotyping at baseline (control session). During the inhibition session, participants received MDZ 1 mg after fluvoxamine intake (50 mg, two doses 12 h and 2 h before MDZ administration) and voriconazole intake (400 mg, one dose 2 h before MDZ administration) [24]. MDZ 1 mg was also received after a pretreatment with the inducer rifampicin (600 mg, one tablet every evening for seven days until the evening before administration of MDZ) (induction session). Urine samples were collected over 8 h after MDZ administration at each session. Venous blood samples (3 mL) were collected before (time 0) as well as 0.5, 1, 2, 3, 4, 6, and 8 h after probe drug administration. Breakfast and lunch were served approximately 1 h and 4 h, respectively, after the blood sampling. The trial is registered at http://www.clinicaltrials.gov (NCT01731067).

### 2.2. Quantification of Exogenous and Endogenous Probes

Analyses of MDZ and OH-MDZ in plasma were performed using a single reverse-phase high-performance liquid chromatography coupled with tandem mass spectrometry (LC-MS/MS) method as described in the study of Bosilkosvka et al. [25].

Urinary DCA and 1β-OH-DCA were quantified according to the method described by Hayes et al. [21] with some modifications. The LC-MS/MS quantification of DCA and DCA-D4 was performed using calibration standards purchased from Sigma (St. Louis, MO, USA), and from Expert Synthesis Solutions (London, ON, Canada) for 1β-OH-DCA and 1β-OH-DCA-D4. To 300 µL of urine samples, 2 µL of the internal standards (IS) stock solution (3000 ng/mL DCA-D4 and 1β-OH-DCA-D4 in methanol) was added. Then, 225 µL of 0.1 M sodium acetate buffer (pH 5.6), 8 µL of β-glucuronidase/arylsulfatase from Helix pomatia (Roche), and 75 µL of choloylglycine hydrolase from Clostridium perfringens (0.2 U/µL) (Sigma) were are also added before incubation overnight at 37 °C.

After incubation, 300 µL of acetonitrile (ACN) were added, and samples were centrifuged at 4000× *g* for 20 min. The supernatant was evaporated under nitrogen at a reduced volume of less than 600 µL. Samples were acidifed with 750 µL of water with formic acid 0.1%. Then, analytes extraction was performed by Solid Phase Extraction (SPE) using Oasis MAX cartridges (Waters). MAX cartridges were conditioned with methanol (1 mL) and equilibrated with water (1 mL). Samples were passed through the MAX cartridges followed by washing with 1 mL of water. All analytes were finally eluted by isopropanol/ACN (6:4) (1 mL). The eluates were evaporated to dryness under a stream of nitrogen and dissolved in 80 µL of ACN 10% before injection into the LC-MS/MS system. Along with the urine samples (including hydrolysis and SPE), standard solutions of DCA and 1β-OH-DCA were prepared in 0.1 M sodium acetate buffer (pH 5.6) at concentrations between 0.1 and 1000 ng/mL with IS [26,27].

LC-MS/MS analyses were carried out using an Agilent 1290 Infinity series LC system from Agilent (Paolo Alto, CA, USA) coupled to a 6500 QTtrap^®^ triple quadrupole linear ion trap mass spectrometer from AB Sciex equipped with an electrospray ionization (Darmstadt, Germany). Separation was performed with a Kinetex^®^ C18 column (50 × 2.1 mm, 2.6 µm) from Phenomenex (Brechbühler, Switzerland). Analyst software (version 1.6.2) was used for system control, data acquisition, and quantification. A linear gradient was applied with a mobile phase composed of (A) water containing 0.1% acetic acid and (B) ACN containing 0.1% acetic acid. Gradient elution was performed at a 500 µL/min flow rate as follows: 0–1.0 min 28% B, 1.0–4.5 from 28% to 30% B, 4.5–7.5 from 30% to 55% B, 7.5–7.6 min from 55% to 90% B, 7.6–8.6 min 90% B, 8.6–8.7 min from 90% to 28% B, and 8.7–10 min 28% B. The injection volume was set at 10 µL. Detection of analytes was obtained in negative mode detection using multiple reaction monitoring (MRM). Most of the unconjugated bile acids, such as DCA and 1β-OH-DCA, do not fragment when subjected to collision-induced dissociation. Therefore, we used a MS/MS transition without fragmentation as performed in previous studies [28,29,30]. Values of QTrap parameters were as follows: curtain gas = 40 psi, collision gas = high, IonSpray voltage = 4500 V, temperature = 550 °C, ion source gas 1 = 60 psi, ion source gas 2 = 60 psi. The MRM transitions for each analyte and IS, and their corresponding optimum MS parameters are described below in Table 1. Accuracy of quality control samples was within 85% and 115% and both intra-day and inter-day variabilities did not exceed 15%.

### 2.3. Statistical Analysis

The pharmacokinetic parameters of MDZ were obtained by standard noncompartmental methods using WinNonlin version 6.2.1 (Pharsight, Mountain View, CA, USA). The following MDZ metrics were measured:

- Oral midazolam clearance (oral MDZ CL)
- 1′-hydroxy-midazolam AUC_0__–8_ to midazolam AUC_0__–8_ ratio (AUC_8_ OH-MDZ/AUC_8_ MDZ)
- 1′-hydroxy-midazolam to midazolam metabolic ratio at 1 and 2 h (OH-MDZ/MDZ MR)
- Midazolam maximum blood concentration (MDZ C_max_).

Inhibition ratios were calculated as follows [31]:

(1) $$\mathrm{Inhibition}\;\mathrm{ratio}\;=\;\frac{{\mathrm{Metric}}_{\mathrm{after}\;\mathrm{inhibition}}}{{\mathrm{Metric}}_{\mathrm{before}\;\mathrm{inhibition}}}.$$

For example, the inhibition ratio of the 1β-OH-DCA/DCA UMR was calculated as below:

(2) $$\mathrm{Inhibition}\;\mathrm{ratio}\;\mathrm{UMR}\;1\beta -\mathrm{OH}-\mathrm{DCA}/\mathrm{DCA}\;=\;\frac{\mathrm{UMR}\;1\beta -\mathrm{OH}-\mathrm{DCA}/{\mathrm{DCA}}_{\mathrm{after}\;\mathrm{inhibition}}}{\mathrm{UMR}\;1\beta -\mathrm{OH}-\mathrm{DCA}/{\mathrm{DCA}}_{\mathrm{before}\;\mathrm{inhibition}}}$$

Similarly, induction ratios were calculated as follows [31]:

(3) $$\mathrm{Induction}\;\mathrm{ratio}\;=\;\frac{{\mathrm{Metric}}_{\mathrm{after}\;\mathrm{induction}}}{{\mathrm{Metric}}_{\mathrm{before}\;\mathrm{induction}}}.$$

The results are presented as geometric means with 95% confidence intervals. Comparisons of intersessions values were performed using a Wilcoxon matched-pairs signed rank test. Pearson’s correlation coefficients were calculated between the log-transformed 1β-OH-DCA/DCA and MDZ pharmacokinetic parameters. All statistical analyses were performed using GraphPad Prism 8.0.1 software (San Diego, CA, USA). *p* ≤ 0.05 was considered statistically significant.

## 3. Results

### 3.1. Subjects

Ten healthy male volunteers were enrolled and completed the study. They were aged between 20 and 36 years of age (median age: 23) with a body mass index (BMI) between 19.9 and 24.4 (median BMI: 22.0) (see the research article by Bosilkovksa et al. [24] for more details).

### 3.2. Exogenous and Endogenous CYP3A Phenotyping Metrics

**Figure 2:**
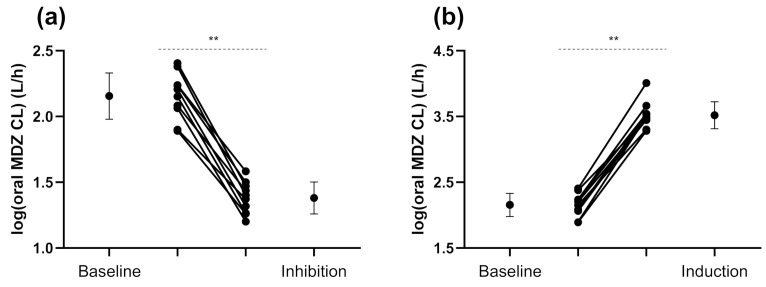
Oral midazolam clearance in ten mal volunteers who received 1 mg midazolam orally at baseline and after (**a**) inhibition by voriconazole and fluvoxamine, and (**b**) induction by rifampicin. Error bars represent the standard deviations of log-transformed oral midazolam clearance and mean values are displayed with a circle. CL, clearance; MDZ, midazolam. ** *p* < 0.01

Oral MDZ CL decreased significantly after a dose of fluvoxamine (50 mg) 12 h prior to MDZ administration, followed by a second dose of fluvoxamine (50 mg) taken concomitantly with voriconazole (400 mg) 2 h before MDZ administration (Figure 2).

All pharmacokinetic parameters of MDZ before and after the ingestion of inhibitors and the inducer are presented in Table 2.

MDZ CL decreased significantly by 83% (inhibition ratio = 0.17-fold, 95% confidence interval (CI) 0.13–0.22, *p* < 0.01) after the ingestion of CYP3A inhibitors. In the case of CYP3A induction by rifampicin, oral MDZ CL was significantly increased by 23.1-fold (95% CI 17.3–30.7, *p* < 0.01). The magnitude of drug–drug interactions (DDIs) was less pronounced when the metabolite OH-MDZ is taken into account. For instance, for OH-MDZ/MDZ MR at 1 h, we measured an inhibition ratio of 0.45 (95% CI 0.36–0.56, *p* < 0.01) and an induction ratio of 5.1 (95% CI 4.2–6.3, *p* < 0.01).

**Figure 3:**
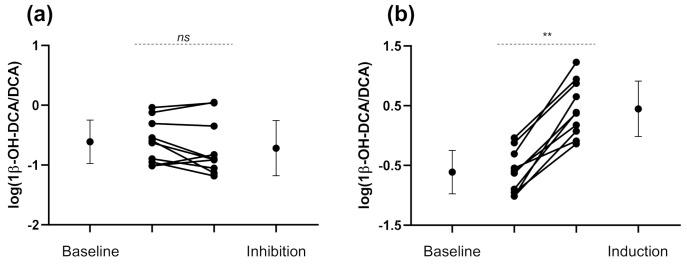
Eight-hour 1β-hydroxy-deoxycholic acid/deoxycholic acid individual urinary metabolic ratios at baseline and after (**a**) the inhibition by voriconazole and fluvoxamine, and (**b**) induction by rifampicin. Error bars represent the standard deviations of log-transformed 1β-hydroxy-deoxycholic acid (1β-OH-DCA/DCA) and mean values are displayed with a circle. 1β-OH-DCA/DCA, 1β-hydroxy-deoxycholic acid/deoxycholic acid. ** *p* < 0.01

Based on in vitro evidence that DCA is a substrate for CYP3A4, the oral administration of CYP3A inhibitors is expected to decrease the UMR 1β-OH-DCA/DCA. Such results were observed but were not significant, as shown in Figure 3 and Table 2 (inhibition ratio = 0.78, 95% CI 0.52–1.2, *p* > 0.05).

Similarly, fluvoxamine and voriconazole caused a non-significant decrease in 1β-OH-DCA urinary excretion of 38% (inhibition ratio = 0.62, 95% CI 0.26–1.5, *p* > 0.05). However, the urinary excretion of DCA was not increased following CYP3A inhibition as theoretically expected (inhibition ratio = 0.79, 95% CI 0.40–1.6, *p* > 0.05).

The endogenous ratio was 11.4-fold higher following rifampicin intake, a CYP3A inducer, compared to baseline (95% CI 6.3–20.7, *p* < 0.01) (Figure 3 and Table 2). 1β-OH-DCA and DCA urinary excretion over 8 h were also significantly increased and decreased, respectively, during the CYP3A4 induction session, as described in Table 2.

### 3.3. Correlations

Table 3 shows the results regarding the Pearson’s correlation coefficients between the log-transformed 1β-OH-DCA/DCA UMR and the log-transformed MDZ metrics, including oral CL, C_max_, AUClast ratio, and MR at 1 and 2 h, for each subject at baseline, after CYP3A inhibition, and after CYP3A induction (sessions measured combined or separately).

Among them, the log-transformed oral MDZ CL and C_max_ correlated significantly with the log-transformed 1β-OH-DCA/DCA UMR at baseline (*r* = 0.652 and *r* = −0.652, respectively, *p* = 0.041). The log-transformed 1β-OH-DCA/DCA UMR correlated also significantly with the log-transformed MDZ CL and MR at 1 h after MDZ intake during the induction session (*r* = 0.678, *p* = 0.031 and *r* = 0.720, *p* = 0.019, respectively), whereas no significant correlations were found following inhibition. When taking into account all sessions simultaneously, the log-transformed 1β-OH-DCA/DCA UMR showed a significant correlation with all MDZ metrics (*p* < 0.0001).

**Figure 4:**
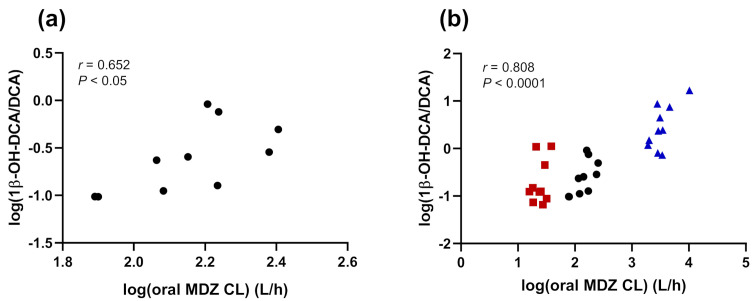
Correlation between log-transformed 1β-hydroxy-deoxycholic acid/deoxycholic acid urinary metabolic ratio and oral midazolam clearance after (**a**) the administration of midazolam alone and after (**b**) the administration of midazolam alone and after pretreatment with voriconazole/fluvoxamine or rifampicin. Black circles, baseline; CYP3A4-inhibited phase, red square; CYP3A4-induced phase, blue triangles. 1β-OH-DCA/DCA, 1β-hydroxy-deoxycholic acid/deoxycholic acid; CL, clearance; MDZ, midazolam.

Figure 4a shows the significant relationship between the log-transformed 1β-OH-DCA/DCA UMR and the log-transformed oral MDZ CL in participants at baseline only. Figure 4b displays the significant correlation between the log-transformed 1β-OH-DCA/DCA UMR and the log-transformed oral MDZ CL in healthy volunteers at baseline, following inhibition with fluvoxamine/voriconazole and induction with rifampicin.

## 4. Discussion

### 4.1. CYP3A Induction

Following one week of treatment with 600 mg of rifampicin, a significant 11.4-fold increase in the endogenous metric 1β-OH-DCA/DCA in human urine compared to baseline was observed. These results confirmed the observations made by Hayes et al. [21], who reported a 7-fold higher ratio in the urine of a carbamazepine-treated patient compared to a control sample. MDZ metrics were also effective in capturing changes in CYP3A activity among participants who received an inducer. For instance, oral MDZ CL and plasma OH-MDZ/MDZ MR at 1 h were significantly increased by 23.1-fold and 5.1-fold, respectively, following rifampicin-mediated induction. The magnitude of CYP3A induction reflected by the endogenous 1β-OH-DCA/DCA UMR was lower compared to oral MDZ CL but higher than plasma 1-OH-MDZ/MDZ MR at 1 h. The greater inter-individual variability observed in the endogenous UMR 1β-OH-DCA/DCA compared to MDZ metrics must be considered with caution and needs to be assessed in future studies with sufficient sample sizes. In particular, extrinsic and intrinsic factors potentially influencing this endogenous metric need to be investigated (e.g., circadian rhythm, pathological state, or fed/fasting conditions) [32,33,34]. The 6β-hydroxycortisol/cortisol UMR is another common endogenous CYP3A metric with high inter-individual variability [35]. Shibasaki et al. [36] demonstrated that the inter-individual variability was lower when measuring the formation clearance (CL_f_) of 6β-hydroxycortisol compared to the UMR, which is likely affected by the variability in the renal CL of cortisol. Therefore, it may also be relevant to test the CL_f_ of 1β-OH-DCA from DCA in future studies.

Interestingly, even when analyzed individually, DCA and 1β-OH-DCA showed significant down- and up-regulation, respectively, during the induction session. Such a pattern was not necessarily followed by other endogenous CYP3A substrates. For instance, plasma concentration of cholesterol, a CYP3A substrate metabolized into 4β-hydroxy-cholesterol, does not appear to decrease, as expected, upon drug induction [37,38]. In the research article of Kasichayanula et al. [38], the plasma concentration of cholesterol even showed a tendency to increase following the administration of rifampicin 600 mg over two weeks. As a result, the ability of plasma 4β-OH-cholesterol/cholesterol to highlight CYP3A induction may be weaker in comparison with other CYP3A4 metrics [16].

### 4.2. CYP3A Inhibition

Fluvoxamine, a moderate CYP3A inhibitor, was ingested 12 h and 2 h prior to MDZ administration, while voriconazole, a potent CYP3A inhibitor, was received as a single dose 2 h before MDZ administration [39,40]. No significant effect on the endogenous metric 1β-OH-DCA/DCA was observed following inhibitors intake. Urinary 1β-OH-DCA/DCA ratio and DCA excretion over 8 h were decreased by around 20% and urinary excretion of 1β-OH-DCA over 8 h was decreased by approximately 40% (all *p* > 0.05). In contrast, MDZ CL decreased significantly by more than 80% following the intake of CYP450 inhibitors. Unlike exogenous probes ingested simultaneously with inhibitors, endogenous metabolites have baseline levels in urine and/or plasma at the time of inhibition. In that case, maximal capture of the inhibition would occur after at least four times the elimination half-life of the endogenous metabolite in order to fully eliminate baseline levels beforehand [41]. The elimination half-life of 1β-OH-DCA is unknown, but it is likely that the CYP3A inhibition duration in this study (12 h) is not long enough to measure significant changes in the 1β-OH-DCA/DCA UMR.

DCA excretion over 8 h was decreased (rather than increased as expected) following inhibitors intake, demonstrating the absence of impact of the modulation on this biomarker in this clinical trial. In this study, enzymatic hydrolysis was performed in order to deconjugate DCA and 1β-OH-DCA, including the predominant amidated conjugates using choloylglycine hydrolase. As performed by Hayes et al. [21], it might be relevant to evaluate whether chemical solvolysis to complete desulfation could help to adjust for these inconsistent results.

Interestingly, in the manuscript of Shin et al. [37], the three endogenous CYP3A substrates cortisol, cortisone, and cholesterol were also illogically down-regulated during the inhibition session (ketoconazole 400 mg once daily for four days) in both plasma and urine samples. Underlying mechanisms related to these observations are unclear. The decreased endogenous synthesis of these steroid hormones by the adrenal glands or increased metabolism may occur to compensate the CYP3A modulation [42,43]. Luo et al. [43] made the same observations regarding cortisol urinary excretion over 24 h, which was not increased following the oral administration of clarithromycin 250 mg twice daily for five days. In contrast, in the same study, the area under the concentration-time curve of plasma endogenous cortisol was significantly higher (+19%, *p* = 0.04) after CYP3A inhibition compared to baseline conditions, but there were no explanations for such discrepancies between plasma and urine [43]. Conversely, the endogenous metabolites, i.e., urinary 6β-hydroxy-cortisol, urinary 6β-hydroxy-cortisone, and plasma 4β-hydroxy-cholesterol, always seem to be consistently down-regulated during the inhibition phase [16,37,43].

### 4.3. Correlations

In addition to providing a real-time snapshot of factors influencing the enzymatic pathway of interest (e.g., inhibition or induction), a probe must correlate with an exogenous reference probe drug [15]. In this clinical trial, the relationships between the urinary endogenous 1β-OH-DCA/DCA ratio and several MDZ metrics at baseline, after CYP3A inhibition, and after CYP3A induction were examined. Despite the small size of the study, the significant correlations observed at baseline with oral MDZ CL and C_max_ are very promising. It indicates that the endogenous 1β-OH-DCA/DCA UMR is a potential metric to phenotype CYP3A enzymes, which could possibly replace the need to administer MDZ. Moreover, Pearson’s correlation coefficients were relatively high between the 1β-OH-DCA/DCA ratio and oral MDZ CL (*r* = 0.652, *p* = 0.041) and C_max_ *(r* = −0.652, *p* = 0.041), indicating a strong relationship. Interestingly, during the induction phase, significant correlations were also observed between the bile acid ratio and MDZ CL (*r* = 0.678, *p* = 0.031), and MR at 1 h (*r* = 0.720, *p* = 0.019), showing a similar capacity to reflect CYP3A induction between the different metrics. Such correlations were not observed during the inhibition session, which is likely due to the lack of inhibitory effects observed on DCA and 1β-OH-DCA, as described previously, compared to MDZ. When data from control, inhibition, and induction sessions were used concomitantly, the log-transformed 1β-OH-DCA/DCA UMR correlated significantly with all MDZ metrics (all *p* < 0.0001), confirming the ability of this endogenous marker to predict CYP3A activity when altered by DDIs, in particular CYP3A induction.

In most studies, the other endogenous metrics, e.g., urinary 6β-hydroxy-cortisol/cortisol ratio, showed no or weak correlation with MDZ metrics under basal conditions [15]. For instance, Luo et al. [43] demonstrated no correlation between urinary 6β-hydroxy-cortisol/cortisol ratio and MDZ MR after oral administration (single dose of 7.5 mg) in 24 healthy volunteers. In the study by Lee et al. [18], the correlation between MDZ CL (measured after a single intravenous dose MDZ 1 mg) and 6β-hydroxy-cortisol/cortisol MR was significant but low (Pearson *r* = 0.278, *p* < 0.01) in 100 healthy subjects. Regarding plasma 4β-hydroxy-cholesterol, significant but relatively weak correlations have been described with MDZ CL at baseline, as summarized in two reviews dedicated to 4β-hydroxy-cholesterol [16,44]. Until recently, the highest correlation was reported by Tomalik-Scharte et al. [45], with Spearman’s rank correlation coefficients of 0.348 observed between plasma 4β-hydroxy-cholesterol/cholesterol and MDZ CL after the intravenous administration of MDZ in 50 healthy volunteers. However, lately, a stronger correlation (*r* = 0.69) has been demonstrated between MDZ MR (single oral dose of 2 mg) and plasma 4β-hydroxy-cholesterol in 73 study participants (from a nondiabetic and diabetic population) [46].

Globally, correlations between endogenous and exogenous probes are subject to large discrepancies between the different studies. These differences can be related to many factors: the sample size used, the endogenous metric used (normalized versus not normalized, MR versus formation CL) and, very importantly, the exogenous metric used—in particular, its mode of administration (oral versus intravenous). Since intestinal and hepatic CYP3A activity are not correlated within the same subject, it is important to use the appropriate probe according what needs to be characterized. In the case of exogenous probes, oral probes will be used to reflect hepatic and intestinal activity, and an intravenous probe will be used to reflect hepatic activity only [16,44]. As described by Penzak et al. [44], endogenous biomarkers represent unusual probes because it is not known whether they can reflect intestinal metabolism, as they are not administered either orally or intravenously. Some authors assumed that endogenous CYP3A markers will only phenotype hepatic activity, and for these reasons, they used intravenous MDZ to establish correlations [17]. However, as most of the drugs are administered orally, we believe that an ideal endogenous probe should also reflect CYP3A intestinal metabolism. In this context, bile acids are promising CYP3A biomarkers, since they are actively reabsorbed in the distal ileum by the apical sodium-dependent bile acid transporter [47]. DCA is a secondary bile acid produced through 7-dehydroxylation by gut bacteria of cholic acid (CA), which is a primary bile acid synthesized from cholesterol into the liver [22]. Then, DCA is likely recycled in the enterohepatic system, where its biotransformation into the tertiary bile acid 1β-OH-DCA may occur via intestinal and hepatic CYP3A [23]. Thus, it appears appropriate to correlate the urinary 1β-OH-DCA/DCA ratio with MDZ metrics measured after oral administration of MDZ (rather than intravenous administration).

## 5. Conclusions

In this clinical trial, we assessed the suitability of two bile acids, DCA and its metabolite 1β-OH-DCA, to substitute MDZ in order to predict CYP3A activity. Indeed, sub-therapeutic doses of oral MDZ (1 mg), as used in this clinical trial, help to avoid possible adverse effects, but phenotyping using exogenous probes still involves cost, reconditioning, and/or manufacturing (and therefore, a risk of error). In addition, the administration of non-therapeutic probe drugs can cause ethical challenges in vulnerable individuals, such as pregnant women or children [48]. For these reasons, the use of an endogenous marker to phenotype CYP3A activity appears more convenient.

In this study, additional clinical data were provided for the urinary endogenous 1β-OH-DCA/DCA ratio, which is a promising metric for CYP3A phenotyping first evidenced by Bodin et al. [20] a few years ago. Significant correlations were obtained between the urinary 1β-OH-DCA/DCA ratio and some MDZ metrics at baseline and after induction.

This biomarker can also accurately detect changes in CYP3A activity due to enzyme induction by rifampicin in ten healthy male volunteers. In contrast, we believe that CYP3A inhibitors were not administered long enough to demonstrate significant changes in the urinary bile acids levels. Further studies are needed in this regard to validate the use of the 1β-OH-DCA/DCA couple as an endogenous CYP3A probe. Comparison with other endogenous CYP3A probes may also be relevant to study in larger sample sizes. Potentially, a combination with other endogenous CYP3A probes may be considered to improve the prediction of enzyme activity using multivariate models. Additional investigations appear also required to understand in more detail the role of intestinal CYP3A in the metabolism of DCA. The limitations of the current study are that only male volunteers were included and that subjects were not genotyped for CYP3A4 and CYP3A5 enzymes. Genotyping was considered irrelevant due to the small sample size and low frequency of the alleles of interest in the Caucasian population (e.g., CYP3A4*22 and CYP3A5*1) [49]. In a previous study including forty-two healthy volunteers, we observed non-significant differences in the 1β-OH-DCA/DCA UMR between the CYP3A poor, intermediate, and extensive metabolizers (manuscript submitted for publication). In vitro, CYP3A5 appears also minimally involved in the hydroxylation of DCA [21]. Therefore, it seems important to study this pathway in larger in vivo study cohorts to determine whether this endogenous biomarker can serve as a selective probe for CYP3A4 phenotyping or whether it is a CYP3A endogenous probe.

## Figures and Tables

**Table 1:** LC–MS/MS settings for the analytes and their respective internal standards (IS).

Compound	MRM	Collision Energy (V)	Declustering Potential (V)	Cell Exit Potential (V)
**DCA**	391.2 → 391.2	−20	−160	−17
**DCA-D4**	395.2 → 395.2	−20	−160	−17
**1β-OH-DCA**	407.2 → 407. 2	−18	−200	−29
**1β-OH-DCA-D4**	411.3 → 411.3	−18	−200	−29

**Table 2:** Pharmacokinetic parameters of endogenous and exogenous probes before and after the ingestion of inhibitors and the inducer, including inhibition and induction ratios.

	Baseline	Inhibition	Inhibition Ratio	Induction	Induction Ratio
**UMR** **1β-OH-DCA/DCA**	0.25 (0.13–0.45)	0.19 (0.09–0.41)	0.78 (0.52–1.2)	2.8 (1.3–6.0)	11.4 ** (6.3–20.7)
**Urinary DCA excretion (µg/8 h)**	63.3 (27.6–145.1)	50.2 (22.7–110.8)	0.79 (0.40–1.6)	22.1 (9.2–52.8)	0.35 * (0.13–0.92)
**Urinary** **1β-OH** **-DCA excretion (µg/8 h)**	15.5 (5.8–41.3)	9.6 (4.1–22.5)	0.62 (0.26–1.5)	61.8 (30.9–123.3)	4.0 * (1.4–11.0)
**Oral MDZ CL (L/h)**	143.0 (107.1–191.0)	24.0 (19.7–29.3)	0.17 ** (0.13–0.22)	3296 (2351–4622)	23.1 ** (17.3–30.7)
**AUC_8_ OH-MDZ/AUC_8_ MDZ**	0.45 (0.36–0.58)	0.17 (0.14–0.22)	0.38 ** (0.30–0.47)	2.4 (2.0–2.9)	5.4 ** (4.2–6.8)
**OH-MDZ/MDZ MR at 1 h**	0.47 (0.37–0.58)	0.21 (0.17–0.27)	0.45 ** (0.36–0.56)	2.4 (2.0–2.8)	5.1 ** (4.2–6.3)
**OH-MDZ/MDZ MR at 2 h**	0.44 (0.33–0.59)	0.22 (0.17–0.28)	0.49 ** (0.38–0.65)	2.6 (2.2–3.0)	6.0 ** (4.3–8.3)
**MDZ C_max_ (ng/mL)**	3.1 (2.3–4.2)	11.7 (9.2–14.9)	3.7 ** (2.7–5.2)	0.15 (0.10–0.23)	0.05 ** (0.03–0.07)

All data are presented as geometric means with 95% confidence intervals. AUC, area under the plasma concentration–time curve; CL, clearance; C_max_, maximum plasma concentration; DCA, deoxycholic acid; MDZ, midazolam; MR, metabolic ratio; OH-MDZ, 1′-hydroxy-midazolam; UMR, urinary metabolic ratio; 1β-OH-DCA, 1β-hydroxy-deoxycholic acid. * *p* < 0.05; ** *p* < 0.01.

**Table 3:** Correlation of log-transformed urinary 1β-OH-DCA/DCA ratio with log-transformed midazolam-based CYP3A4 metrics at baseline, after inhibition, and after induction.

	Baseline		Inhibition		Induction		All Sessions	
	*r*	*p*	*r*	*p*	*r*	*p*	*r*	*p*
**Oral MDZ CL (L/h)**	0.652	0.041	0.336	0.343	0.678	0.031	0.808	<0.0001
**AUC_8_ OH-MDZ/AUC_8_ MDZ**	0.275	0.442	0.172	0.635	0.567	0.088	0.781	<0.0001
**OH-MDZ/MDZ MR at 1 h**	0.331	0.351	−0.040	0.914	0.720	0.019	0.786	<0.0001
**OH-MDZ/MDZ MR at 2 h**	0.242	0.500	0.215	0.550	0.500	0.141	0.790	<0.0001
**MDZ C_max_ (ng/mL)**	−0.652	0.041	−0.491	0.150	−0.456	0.185	−0.821	<0.0001

AUC, area under the plasma concentration–time curve; CL, clearance; C_max_, maximum plasma concentration; DCA, deoxycholic acid; MDZ, midazolam; MR, metabolic ratio; OH-MDZ, 1′-hydroxy-midazolam; 1β-OH-DCA, 1β-hydroxy-deoxycholic acid.

## Data Availability

The data presented in this study are available on request from the corresponding author.
